# GATC: a genetic algorithm for gene tree construction under the Duplication-Transfer-Loss model of evolution

**DOI:** 10.1186/s12864-018-4455-x

**Published:** 2018-05-09

**Authors:** Emmanuel Noutahi, Nadia El-Mabrouk

**Affiliations:** Département d’Informatique et de Recherche Opérationnelle, Université de Montréal, Montréal, Canada

**Keywords:** Gene tree, Genetic algorithm, Phylogeny, Reconciliation, Gene duplication, Horizontal gene transfer

## Abstract

**Background:**

Several methods have been developed for the accurate reconstruction of gene trees. Some of them use reconciliation with a species tree to correct, *a posteriori*, errors in gene trees inferred from multiple sequence alignments. Unfortunately the best fit to sequence information can be lost during this process.

**Results:**

We describe GATC, a new algorithm for reconstructing a binary gene tree with branch length. GATC returns optimal solutions according to a measure combining both tree likelihood (according to sequence evolution) and a reconciliation score under the Duplication-Transfer-Loss (DTL) model. It can either be used to construct a gene tree from scratch or to correct trees infered by existing reconstruction method, making it highly flexible to various input data types. The method is based on a genetic algorithm acting on a population of trees at each step. It substantially increases the efficiency of the phylogeny space exploration, reducing the risk of falling into local minima, at a reasonable computational time. We have applied GATC to a dataset of simulated cyanobacterial phylogenies, as well as to an empirical dataset of three reference gene families, and showed that it is able to improve gene tree reconstructions compared with current state-of-the-art algorithms.

**Conclusion:**

The proposed algorithm is able to accurately reconstruct gene trees and is highly suitable for the construction of reference trees. Our results also highlight the efficiency of multi-objective optimization algorithms for the gene tree reconstruction problem. GATC is available on Github at: https://github.com/UdeM-LBIT/GATC.

**Electronic supplementary material:**

The online version of this article (10.1186/s12864-018-4455-x) contains supplementary material, which is available to authorized users.

## Background

Most biological discoveries can only be made in the light of evolution. In particular, functional annotation of genes is usually deduced from the orthology and paralogy relation between genes, which is inferred from the comparison of a gene tree with a species tree. Therefore, phylogenetic tree reconstruction is an important component of most bioinformatic pipelines. In this paper, we focus on the reconstruction of gene trees.

Standard phylogenetic tools are based on maximum likelihood (ML) or bayesian methods reconstructing a tree from gene sequences (e.g. PhyML [1], RAxML [2], MrBayes [3], PhyloBayes [4]). However, for a variety of reasons due, not only to technical limitations but also to the data itself (sequences too close to each other or conversely too divergent), sequence-only methods often do not allow to confidently discriminate one tree from another.

To address this limitation, more recent gene tree reconstruction methods, designated here as *integrative methods*, include information from the species tree. The idea is to consider, in addition to a maximum likelihood value measuring the fitness of a tree to a sequence alignment (according to a model of sequence evolution), a measure reflecting the evolution of a whole gene family through gene gain and loss. A standard measure of fitness between a gene tree and a species tree is computed in terms of a “reconciliation” score. In a probabilistic framework, the reconciliation score corresponds to the probability density of the gene tree given some parameters (rates of events and branch lengths) under a birth-death and gain model of evolution. For the Most Parsimonious Reconciliation model (MPR), this score corresponds to the optimal number of gene gain and loss events, weighted by their costs, explaining the incongruence between a gene tree and a species tree.

Most integrative methods for gene tree reconstruction assume a simplified model of gene family evolution where gene gain events are reduced to gene duplication (e.g. TreeBeST [5], TreeFix [6], ProfileNJ [7], NOTUNG [8], SPIMAP [9], Giga [10]). In fact, the MPR problem for the Duplication-Loss (hereafter denoted DL) model of gene family evolution is linear-time solvable [11]. By introducing horizontal gene transfer (HGT) events, the Duplication-Transfer-Loss (DTL) model becomes NP-hard in general if time consistency is required for inferred events (unless the species tree is fully dated) [12–14]. However the MPR problem for the DTL model, with an undated species tree, can still be computed in polynomial time if the time consistency requirement is relaxed [15–17]. Due to this reasonable time-complexity, some recent phylogenetic softwares have extended the gene family evolution model to transfers (MowgliNNI [18], ecceTERA [19], TreeFix-DTL [20]). Continuous effort is also made for developing fast probabilistic frameworks capturing HGT events (see [21] for a review of these models).

Integrative methods report gene trees with better accuracy compared with sequence-only methods [18, 20, 22, 23], but they still leave space for improvement, both on tree quality and on computation time. In fact, most of them rely on a two-steps approach, first computing a tree with the best fit to the sequences, and then exploring a tree space surrounding the initial tree to select one minimizing the considered reconciliation distance. From an accuracy point of view, this two step methodology does not guarantee that the output tree optimizes both the likelihood given the sequence alignment, and the reconciliation measure, as the best fit to the sequences may be lost at the second step. In addition, by considering a single tree at a time, the risk of ignoring a large part of the tree space and falling into a local minimum is high. Other integrative methods (see for example PhylDog [24] and PrIME-DLTRS [23]) compute the joint likelihood associated with a substitution model and DTL event rates, given a fixed, dated and utrametric species tree and a gene family alignment. They use tree exploration heuristics similar to those found in sequence-only programs for phylogenetic tree reconstruction to explore the solution space, often in a bayesian-MCMC framework. These methods come at a high computational cost, especially when HGT events are considered, and they are still subject to the risk of being stuck in a local optimum.

In this paper, we present *GATC* (*G*enetic *A*lgorithm for gene *T*ree *C*onstruction), a new software for gene tree reconstruction under the DTL model that can take as input completely unresolved, partially unresolved or fully resolved trees, and outputs a tree minimizing a measure combining both tree likelihood (according to sequence evolution) and a reconciliation score. In other words, it can either be used as a two-step correction method, when input trees are the output of other phylogenetic methods, or as a one-step method resolving a full polytomy (star tree) in a way optimizing fit to both the species tree and the sequences.

With GATC, we explore a new methodological framework based on a Genetic Algorithm (GA), a global search metaheuristic that mimics biological evolution [25]. The ability of GAs to find near-optimal solutions quickly, even for complex models and data makes them suitable for the problem of phylogenetic inference. In fact, the GA methodology has been previously applied to the phylogenetic inference problem, starting with Matsuda in 1996 [26] using a maximum likelihood criterion, Lewis [27] who introduced a subtree swap crossover operator, and other more recent algorithms (e.g. self-adaptive GA [28], Ga-mt [29], METAPIGA [30], GARLI [31]). However, all these algorithms are solely based on sequence information and, as discussed above, are often error-prone in the case of gene tree reconstruction. To our best knowledge, GAs have never been applied to species tree-aware gene tree reconstruction, although the technique is suitable to the resolution of Multi-Objective Optimization Problems (MOOP).

To measure the performance of GATC, we compared it to current state-of-the-art softwares on a dataset of simulated cyanobacterial phylogenies. Our results show that GATC is more accurate than existing methods, suggesting that it substantially increases the efficiency of the phylogeny space exploration. We also evaluated GATC’s ability to infer accurate homology relationships between genes on a standardized, manually curated, dataset of real trees. The predicted relationships were mostly in agreement with the ones inferred from a reference tree, highlighting the efficiency of the framework.

## Methods

### Notation on trees

All considered trees are rooted unless explicitely stated. A tree is *binary* if all its internal nodes have exactly two children, and *non-binary* otherwise. Unless stated differently, all trees are considered binary.

We denote by *V*(*T*) the nodeset, by *E*(*T*) the edgeset, by $\mathcal {L}(T)$ the leafset and by *r*(*T*) the root of a tree *T*. An edge *e* of *E*(*T*) is written as a pair (*x*,*y*) of two adjacent nodes where *e* is an outgoing edge of *x*. For *e*=(*x*,*y*), *x* is the parent *p*(*y*) of *y*, while *y* is a child of *x*. A node *x* is an *ancestor* of *y*, which is denoted *x*<_*T*_ *y*, if it is on the path from *y* to the root (excluding *y*). In this case, *y* is called a *descendant* of *x*. Similarly, an edge *e*^′^=(*x*^′^,*y*^′^) is an ancestor of an edge *e*=(*x*,*y*) if it is on the path from *y* to the root. Given a node *x*, *T*[*x*] is the subtree of *T* rooted at *x* and $\mathcal {L}(x)$ the leafset of *T*[*x*]. Two subtrees *T*[*x*] and *T*[*y*] are *separated* in *T* if *x*≠*y*, $x \nless _{T}\; y$ and $x \ngtr _{T} \; y$. In this case, $\mathcal {L}(x) \cap \mathcal {L}(y) = \emptyset $, if the leaves of *T* are uniquely labeled by the elements of $\mathcal {L}(T)$.

A *species tree* is a tree *S* with leaves uniquely labeled and $\mathcal {L}(S)$ being a set of species. Likewise, a *gene tree* is a tree *G* with leaves uniquely labeled and $\mathcal {L}(G)$ corresponding to a set of genes where each gene *g* belong to a genome *s*(*g*). We denote by $\overline {G}$ the tree obtained from *G* by replacing each leaf label *g*_*i*_ by its genome *s*(*g*_*i*_). Notice that the mapping $s: \mathcal {L}(G) \rightarrow \mathcal {L}(S)$ does not have to be injective nor surjective. In particular, $\overline {G}$ may have several equally labeled leaves.

**Fig. 1:**
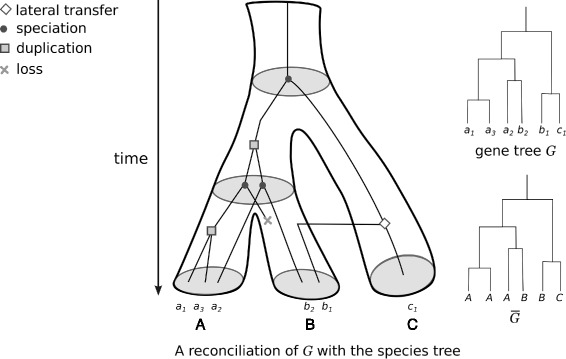
A reconciliation between a gene tree *G* and a species tree. The reconciliation represents a history of the gene family evolution through speciation, gene duplication, gene loss and HGT, in a way that is consistent with the species tree. Both *G* and $\overline {G}$ are shown on the figure

A *reconciliation* of *G* (or similarly $\overline {G}$) with *S* (see Fig. 1) is an extension of *s* from *V*(*G*) to *V*(*S*) with additional labels on each internal node *x* of *G*, describing the type of evolutionary event that has led to *G*[*x*] (duplication, speciation or transfer). *G* can be expanded to include lost genes.

Finally, we refer to the process of removing a leaf *l* and its associated edge (*p*(*l*), *l*) from a tree *T* as the deletion of *l* from *T*.

### Vocabulary of Genetic Algorithms

A Genetic Algorithm (GA) is an algorithmic framework mimicking biological evolution. The vocabulary of a GA is filled with biological metaphors. It begins with a *population* of *individuals* whose *chromosomes or genomes* encode specific solutions to the problem of interest. Performance of theses individuals in solving the problem is measured by their *fitness score*. To avoid confusion, throughout this paper the word “chromosome” will be used solely to designate the data structure of a genetic algorithm, and the word “genome” will be used in its biological meaning to designate the macromolecules containing the genes under study.

At each step, starting from an initial population, a new population is generated using three operators: *selection*, *crossover* and *mutation* [25], which are defined according to the nature of the problem and the encoding of the solution. During selection, the fitness score is used to select individuals for reproduction. Selected candidates are combined using the crossover operator to create new individuals that are then modified by the mutation operator in order to introduce diversity and avoid local optima. With *elitism*, the less fit individuals of the newly obtained population are replaced by the best fit of the previous *generation*, in order to conserve the best solutions found so far. The process described above is repeated through multiple generations, until an optimal solution (chromosome of the best individual) is obtained or a stop criterion is reached.

This natural selection process generally leads to the improvement of the average population fitness over generations. While GAs often converge to an optimal or near optimal solution, their performance mainly depends on the mechanism for balancing two potentially contradictory objectives: keeping the best solutions found so far, and at the same time efficiently exploring the search space for promising solutions.

### The GATC algorithm description

In the rest of the manuscript, we will loosely refer to the tree likelihood given a multiple sequence alignment as *sequence likelihood*.

Given a sequence alignment *D* and a species tree *S*, our objective is to find the gene tree *G* or a set $\mathcal {G}$ of gene trees, with branch length, that are (near) optimal for both the sequence likelihood and the reconciliation score. To solve this problem, our fitness function should reflect both objectives. We will present different ways for computing the fitness score, either by a linear combination of the two scores, or by trying to reach a *pareto optimality*. We start by presenting the general framework of the GA.

#### Solution encoding

A chromosome *σ* is defined as (*G*,*θ*) where *G* is a rooted binary gene tree and *θ* is the set of hyperparameters underlying the evolutionary model. Namely, *θ*=(*λ*,*δ*,*τ*,*e*,*l*,*m*), representing respectively the duplication rate, the loss rate, the transfer rate, the substitution rates across the gene tree edges, branch lengths and the substitution model. Some of these parameters might be kept fixed during the evolutionary process. For example, the substitution model *m* is usually fixed for all generations, whereas duplication, loss and transfer rates can vary when a probabilistic model is used to compute the reconciliation score. When parsimony is preferred, they correspond to fixed event cost. Branch lengths and edge substitution rates are usually optimized during sequence likelihood computation.

For the probabilistic model of reconciliation, the initial values of the hyperparameters *λ*,*δ*,*τ* and *e* are randomly drawn from a uniform distribution, unless explicitely provided. The default substitution model used for nucleotide sequences is the GTR model [32] with a gamma distribution to account for rate variation [33], whereas for proteins, the JTT model [34] with gamma-distributed rates is used.

#### Gene trees in the initial population

When starting trees are available (from any other tree construction method, integrative or not), they can be used as the population of the first generation in our GA. Otherwise the trees of the initial population are generated, either randomly or according to a predefined procedure. GATC implementation allows generating the initial trees from a star tree, using PolytomySolver [35] which outputs the most parsimonious trees for the DL-reconciliation score (but not necessary optimal for the DTL-reconciliation score or the sequence likelihood), or using bootstrapped trees obtained with RAxML [2]. These two methods should be prefered to the initialization with random trees, which can affect the algorithm’s convergence.

Notice that two trees *G*_1_,*G*_2_ such that $\overline {G_{1}}=\overline {G_{2}}$ have the same reconciliation score, and thus if *G*_1_ is a solution of PolytomySolver, minimizing the DL-reconciliation score, then *G*_2_ is also a solution. Therefore, in this case, to increase the initial population of the GA, additional trees can be obtained by permutation of the genes at the leaves of *G*_1_ in a way respecting the mapping function *s*.

#### Computing the sequence likelihood and reconciliation score

To evaluate the fitness of each chromosome *σ*_*i*_,1<*i*<*n*, in a population of size *n*, we first compute a vector $\vec {z}_{i}$ of two components, called the *raw score vector*, containing the sequence likelihood and the reconciliation score. Note that when the objective is to optimize only sequence likelihood, the second component corresponding to the reconciliation score is set to zero.

The sequence likelihood scores *p*(*D*|*G*,*l*,*m*) can be computed using the Felsenstein algorithm [36] and the further computationnal enhancement described by Stamatakis et al. (2004) [37]. In fact, GATC use subroutines from RAxML to compute the sequence likelihood, thus benefiting from both its high computational speed and its large set of substitution models.

As for the reconciliation score, it can be computed under either the probabilistic or MPR model. For the MPR scoring model, we implemented the Bansal algorithm [15] which computes the DTL reconciliation cost between a binary gene tree *G* and a binary species tree *S* in time *O*(|*G*||*S*|). Notice that, as explicit transfer pathways are not specified, a DTL scenario is not necessarily possible as it may violate temporal constraints [14]. In fact, a donating and a receiving species must have co-existed at the time of the transfer. Moreover, in contrast to duplications and losses, HGT are inter-dependent, and can induce contradictory temporal constraints on ancestral species. However, as the reconciliation problem for DTL using undated species tree with the constraint of respecting temporal constraints is NP-hard, the Bansal algorithm remains a good alternative for computing a reasonable DTL reconciliation score. In absence of HGTs, we compute the DL reconciliation score using a linear-time algorithm [11], to speed up calculations.

For the probabilistic scoring model, we have implemented the DTL model first described by Tofigh [38, 39] and used by PrIME-DLRS [23]. It is based on a birth-death model of evolution including rates for gene duplication, transfer and loss that requires discretization of a dated species tree and numerical resolution of ordinary differential equations. We refer the readers to the Supplementary Material of [23] for a thorough description of how the probability density of the reconciliation is computed.

Rather than minimizing the reconciliation score and maximizing likelihood, it is easier to simultaneously minimize both measures. For this reason, we take the negative log value when likelihood is used for any of the two scores. Therefore, it has to be understood that the best adapted individuals will be those with the lowest fitness.

#### Computing the fitness score

Given a raw score vector $\vec {z_{i}}$ for a chromosome *σ*_*i*_, a weight vector $\vec {w}$ and a scaling function *ϕ*, we define the fitness score *f*_*i*_ of *σ*_*i*_ as $f_{i} = \vec {w} \boldsymbol {\cdot }\phi (\vec {z_{i}})$. In other words, *f*_*i*_ corresponds to the weighted sum of the two components of the raw score vector, scaled by a function *ϕ*. The simplest definition of *ϕ* is the identity function $\phi (\vec {z}) = \vec {z}$. An alternative is to standardize each score to a zero-minimum resulting in the following formulation : $\phi \left ({z_{i}^{k}}\right) = {z_{i}^{k}} - \text {min}_{i} (z_{i})^{k}$ for 1≤*k*≤2 and 1≤*i*≤*n*. However, for this latter scaling function, fitness is not comparable between individuals of different generations.

Using the method described above for computing *f*_*i*_ transforms our problem into a single objective minimization problem and is suitable when both components of *z*_*i*_ are log likelihood values, since it is related to the joint weighted probability density for sequences data and reconciliation to the species tree.

When the reconciliation score is computed using parsimony, combining the two scores this way might not be optimal. Instead, we compute a set of *pareto optimal solutions* for this multi-objective optimization problem (MOOP). Several evolutionary based techniques have been developed for MOOP [40]. Here we will use a technique similar to the widely known NSGA (Non-dominated Sorting Genetic Algorithm) [41].

A raw score vector $\vec {z_{i}} = \left ({z^{1}_{i}}, {z^{2}_{i}}\right)$ is said to dominate another vector $\vec {z_{j}} = \left ({z^{1}_{j}}, {z^{2}_{j}}\right)$, denoted as $\vec {z_{i}} \prec \vec {z_{j}}$, iff $\vec {z_{i}} \neq \vec {z_{j}}$ and ${z^{1}_{i}} < {z^{1}_{j}} \;, {z^{2}_{i}} < {z^{2}_{j}}$. We are interested in finding the set of non-dominated solutions called *pareto set* (PS) and denoted as :

$$ PS = \{\sigma_{i}\;|\; \nexists\; \sigma_{j}, \vec{z_{j}} \prec \vec{z_{i}}\} $$

At the end of the GA’s evolutionary process, the pareto set represents the set of pareto optimal solutions. In contrast with classical genetic algorithms, computing the pareto set requires to consider simultaneously a parent population *P*_*i*_ and its offspring $P^{\prime }_{i}$, as optimal solutions from *P*_*i*_ can be lost if we use $P^{\prime }_{i}$ as the population *P*_*i*+1_ of the next generation.

Algorithm 1 illustrates the way fitness is computed for all individuals of a generation. It proceeds in a *wave* fashion, selecting the non-dominated individuals from the population *P*^∗^=*P*_*i*_∪*Pi*′, assigning them a shared fitness score, and then removing them from *P*^∗^. This process is repeated while increasing the fitness score for the individuals in the new waves, until the expected population size per generation is met or there is no non-dominated individuals anymore. In the latter case, the fitness of the remaining individuals is computed as the sum of their *dominance rank* (number of individuals that dominates an individual) and the fitness of the last wave. This process ensures that individuals belonging to the same wave have the same fitness and as such the same probability to reproduce. The *n* individuals with the best fitness constitutes *P*_*i*+1_. Selection, crossover and mutation operators can be applied to *P*_*i*+1_ resulting in offspring $P^{\prime }_{i+1}$.



#### Selection

GATC implements multiple classical selection methods. Individuals can either be selected for crossover using the tournament selector [42] or using the roulette wheel selector which chooses individuals with probability inversely proportionnal to their fitness values (recall that the best indivuals have the smallest fitness value). Alternatively, the random uniform selector can be used, which gives equal reproduction probability to all individuals regardless of their fitness. Selected indivuals are used in the crossover operator to produce the individuals of the next generation.

#### Crossover

In the crossover operators implemented in GATC, two offsprings are created from two parent chromosomes. Each offspring inherits its hyperparameter *θ* from one of its parents, while its gene tree is obtained from the combination of the two parental trees.

Given trees *G*_*i*_ and *G*_*j*_ respectively from parent *σ*_*i*_ and *σ*_*j*_, the first offspring is obtained with the subtree swap crossover operator [27], achieved by the following actions:

1. Select a subtree *G*_*i*_[*x*] from *G*_*i*_ (the root is excluded)
2. Delete all leaves from *G*_*j*_ that are also in $\mathcal {L}(x)$;
3. Regraft *G*_*i*_[*x*] to a random edge of *G*_*j*_ to obtain the offspring tree $G^{\prime }_{j}$.

**Fig. 2:**
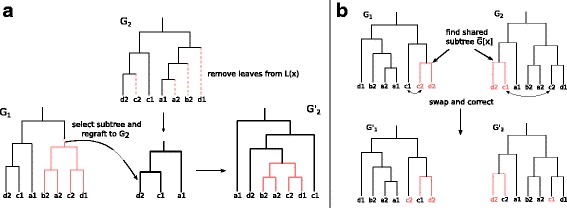
Crossover operator. **a** Subtree swap. A subtree *G*_1_[ *x*] (in red) is pruned from *G*_1_ then regrafted to a random branch of *G*_2_ after deleting from *G*_2_ its leaves that also appear in *G*_1_[ *x*] (shown in dotted lines). To obtain the second child, a similar operation is performed from *G*_2_ to *G*_1_**b** Subtree swap preserving reconciliation. Two subtrees *G*_1_[ *x*] and *G*_2_[ *y*], respectively from *G*_1_ and *G*_2_, such that $\overline {G_{1}[\!x]}=\overline {G_{2}[\!y]}$ are swapped and the remaining leaves are corrected to conserve the same leafset as the parent

The second offspring tree $G^{\prime }_{i}$, is obtained in a similar way by selecting a subtree from *G*_*j*_ and regrafting it in *G*_*i*_. The crossover operator is illustrated on Fig. 2a.

In the special case where the objective is to only optimize the sequence likelihood, under the hypothesis that the reconciliation score is already optimal, this crossover operator is not applicable as it does not preserve the reconciliation score. Instead, the offspring trees are created by exchanging two subtrees *G*_1_[*x*] and *G*_2_[*y*] such that $\overline {G_{1}[x]}$ and $\overline {G_{2}[y]}$ are isomorphic with respect to the labels at their leaves (see Fig. 2b).

#### Mutation

**Fig. 3:**
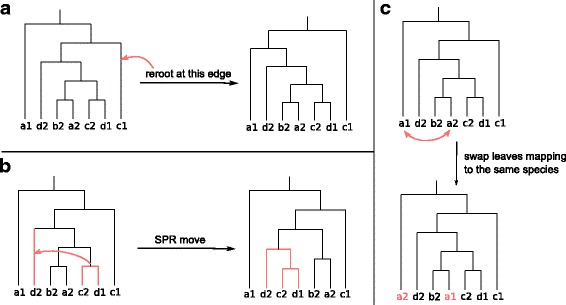
Mutation operator. **a** Re-rooting. The tree is rerooted at a random edge. **b** SPR move. A subtree is pruned from the tree and regrafted to another edge. **c** Mutation preserving reconciliation cost. Two leaves *l*_1_ and *l*_2_ such that *s*(*l*_1_)=*s*(*l*_2_) are swapped. This mutation only alter the sequence likelihood

For a chromosome *σ*_*i*_=(*G*_*i*_,*θ*_*i*_), a mutation is performed either on the tree *G*_*i*_ or on the rates *λ*,*δ*,*τ*,*e* unless their values are fixed. Mutations on the rate parameters consist in drawing a new value from their distribution. On the other hand, a mutation operates on *G*_*i*_ by applying a topological modification. GATC uses SPR (Subtree Pruning and Regrafting) and re-rooting operations (see Fig. 3a-b) to generate a new tree topology. As with the crossover operator, when only sequence likelihood has to be optimized, reconciliation score should be preserved after mutations. For this purpose, mutation are performed by permuting the genes assignment to the leaves of *G*_*i*_ in such a way that only genes belonging to the same species are allowed to switch places (see Fig. 3c).

#### Stop criteria

We proposed several criteria to stop the GA evolution. The simplest ones are to terminate when a maximum number of generations or a time limit are met, or when all individuals converge to a single fitness value. Aside from these criteria, we propose another simple termination criterion called *population-AU criterion* that is based on the use of a reference ML tree. Under this criterion, evolution is stopped when all the individuals in the current population are statistically equivalent to the known ML tree, according to the AU test [43]. This stop criterion allows for a good performance when the objective is restricted to the optimization of sequence likelihood.

## Results and discussion

To measure the efficiency of GATC in reconstructing accurate gene trees, we compared its performance, on a simulated dataset, to four different gene tree reconstruction methods: ecceTERA [19], TreeFix-DTL [20], ProfileNJ [7], MowgliNNI [18] and RAxML [2]. In contrast to RAxML which is a sequence-only method, the former four methods use both sequences and species tree information. We also used GATC to reconstruct the gene trees of three gene families for which reference trees have been proposed [44]. We will entirely focus on evaluating GATC’s performance under the MPR model, as it is our main contribution and also because DTL-reconciliation scores can be computed significantly faster under this model.

### Evaluation on a simulated Cyanobacteria histories dataset

We used the simulated cynobacteria dataset of Szöllosi et al. (2013) [22] publicly available at http://datadryad.org/resource/doi:10.5061/dryad.pv6df. This dataset consists of 1099 gene families from 39 cyanobacteria species along with a well-resolved dated species tree. To construct the dataset, the gene families were retrieved from HOGENOM [45] and multiple alignments were performed on these families with Muscle [46]. For each alignment, an MCMC sample of at least 3000 trees was obtained with PhyloBayes [47] and used to reconstruct an amalgamated tree with ALE [22]. These trees were used to simulate new multiple alignments of artifical sequences under the LG model with a gamma distribution. We refer to [22] for a more detailed description on the construction of the dataset.

From each of the 1099 simulated artificial sequence alignments, we reconstructed an inital tree using RAxML (LG + Gamma, 100 bootstraps). The RAxML trees (with bootstrap values) were used as input for all programs being compared against GATC.

For all programs, we used fixed DTL rates (*λ*=2,*τ*=3,*δ*=1) except for ProfileNJ which supports only a DL model of reconciliation and for which we took *τ*=*∞*. We ran TreeFix-DTL with default parameters and LG + Gamma as model of evolution. As it requires rooted trees, the input RAxML trees were rooted using the mid-point rooting method [48]. MowgliNNI, ecceTERA and ProfileNJ were run with a threshold of 0.7 for the contraction of for weak edges. Note that ProfileNJ and ecceTERA can output several solutions from which users can later select a tree according to some other measure (sequence likelihood for example). In our comparison, we only consider the first solution returned by both methods, as this selection process is not part of the methods, and also removes their running time advantage. We ran GATC with the following parameters : a maximum of 50 generations, a time limit of 90 minutes per gene family, LG + Gamma as the model of evolution and parsimony for DTL-reconciliation. We used the tournament selector as the selection operator and set the crossover and mutation rates to 0.8 and 0.5 respectively (see Additional file 1: Section 1 and Figure S1, for a discussion on the effect of crossover and mutation rates on accuracy). To construct the initial population of the GA, we used PolytomySolver’s resolutions of RAxML trees after contraction of edges with support less than 0.7. In order to keep the GA population size fixed at 30, we randomly removed or duplicated chromosomes from the initial population until its size became 30. We also used the population-AU as additional stopping criterion with the RAxML tree being the known best ML tree and a significance level *α*=0.05. When there were more than one tree in the pareto optimal set, the tree with the lowest DTL-reconciliation score was returned as GATC final solution.

**Fig. 4:**
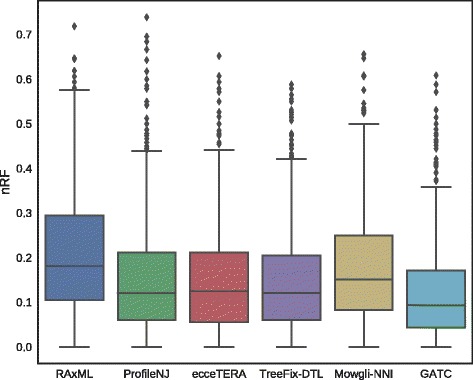
Accuracy of RAxML, ProfileNJ, ecceTERA, TreeFix-DTL, Mowgli and GATC on a dataset of simulated Cyanobacteria histories: we measure the normalized Robinson-Foulds distance of the reconstructed trees to the true gene trees for all 1099 gene families. GATC achieves the best accuracy on the simulated dataset, followed by TreeFix-DTL

We measured the accuracy, defined as the normalized Robinson-Foulds distance between each reconstructed tree and the true tree. As shown in Fig. 4, trees reconstructed with species tree-aware algorithms were more accurate than RAxML’s trees. This result was expected, since it has been shown several times that integration of species tree information usually improves gene trees reconstruction. GATC, in particular, achieves a better accuracy than other methods, due to its improved tree space search efficiency. The algorithm also appears to be robust, in some extent, to errors in the species tree topology (see Additional file 1: Section 2 and Figure S2). However, it should be noted that in order to obtain accurate results, there is a need to allocate a considerable time for the evolution of the GA. As such, the algorithm is much slower, in comparison to ProfileNJ and ecceTERA which can output solutions in a few seconds. To our surprise, ProfileNJ was almost as accurate as the second best method (TreeFix-DTL), although it only supports a DL model of reconciliation and HGT were present in the dataset. It is possible that most edges with weak support were not involved in HGT events, which can explain the observed performance of ProfileNJ.

### Evaluation on an empirical dataset

In an attempt to establish a benchmark for comparing orthology prediction methods, Boeckmann et al. (2011) [44] proposed manually curated “gold standard” gene trees for three well-conserved gene families : the Popeye-domain containing family (POP), the NOX ‘ancestral-type’ subfamily of NADPH oxidases (NOX) and the V-type ATPase beta subunit (VATP).

These gene families have been re-analyzed here to assess the performance of GATC on an empirical dataset. The reference species tree used was obtained from SwissTree [49]. Protein sequences from genomes not found in the species tree were removed and the remaining sequences aligned with Muscle [46]. GATC was used to reconstruct the corresponding trees for each gene family with initial population of trees obtained from bootstrap replicates. We used the same parameters as above except for the DTL events cost, which was changed to: (*λ*=1, *τ*=*∞*,*δ*=1). Here, we prohibit HGT events since they are not expected in the dataset. We also set the maximum number of generations to 300 and the maximum time of evolution to 3h per gene family. For comparison, the average time needed by RAxML to obtain the best ML trees is 2.4h.

**Fig. 5:**
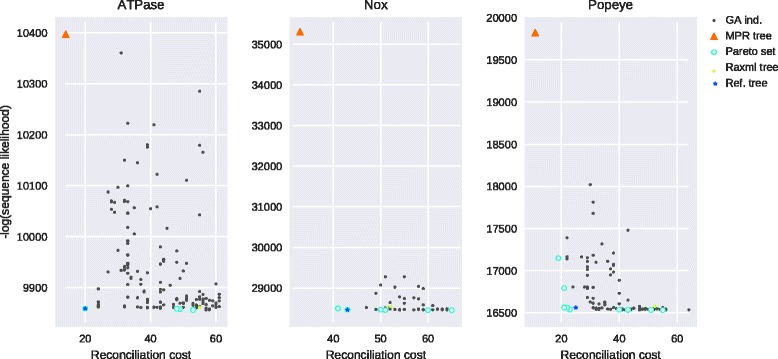
Distribution of individuals’ raw scores during evolution on three “gold standard” gene families. The scores of the ML tree obtained with RAxML, the MPR tree for the DL score, and the reference gene tree of [44] are also shown. Note that for fair compairson, the RAxML tree reconciliation score correspond to the best rooting score, whereas the MPR tree sequence likelihood correspond to the tree with the minimum negative log likelihood in the set of equivalent MPR trees. For the sake of visibility, we increased the size of each data point. The “best tree” is expected to be located in the lower left corner. For the ATPase and Nox families, the reference tree was present in the set of pareto optimal trees returned by GATC. For the Popeye gene families, the reference tree was located in the proximity of a cluster of pareto optimal solutions

In order to measure the accuracy of GATC, we investigated how close the reference trees were to the set of pareto optimal trees. Figure 5 shows the distribution of individuals’ scores, over generations, during the GA evolution for each gene family. We were able to retrieve the reference tree for the NOX and VATP gene families, whereas the reference tree for the POP family was located close to a cluster of pareto optimal trees. From the same figure, it can also be seen that even though the ML and MPR trees theoretically belong to the pareto optimal set of the complete tree space, they are often located far from the desired optimal result.

Since the reference gene tree was not obtained for the POP family, we report the precision and recall of orthologous and parologous genes inference from the solutions returned by GATC, compared to the proposed reference tree (Table 1). Note that GATC only outputs ten trees from the 30 individuals of the final population resulting in four unique trees (see Additional file 1: Figures S4-S7). We computed precision and recall for the two types of gene relationships as follows:

$$Precision = \frac{TP}{TP+FP}\;\;, \;\;Recall = \frac{TP}{TP+FN} $$

where TP corresponds to the number of shared pairs of orthologs/paralogs with the reference tree, FP corresponds to the number of predicted pairs of orthologs/paralogs not present in the reference tree, and FN to the number of missed orthologs/paralogs. As shown on Table 1, the precision and recall for the inferred gene relationships were high for all four solutions. Difference between GATC’s solutions and the reference POP gene family tree (Additional file 1: Figure S3) can mostly be explained by the fact that duplication nodes were often placed lower in the solutions, resulting in fewer number of losses and consequently lower reconciliation scores.

**Table 1:** Comparison between the reference tree of the Popeye family and the pareto optimal trees returned by GATC

	NormRF distance	Orthologs	Paralogs		
		Prec.	Rec.	Prec.	Rec.
Tree 1	0.260	0.763	0.942	0.971	0.871
Tree 2	0.260	0.765	0.941	0.971	0.873
Tree 3	0.087	0.902	0.983	0.992	0.894
Tree 4	0.109	0.829	0.866	0.940	0.922

It is hard to argue whether the proposed reference tree represents the true evolutionary history of the gene family over our pareto optimal solutions. In fact, from Fig. 5, it can be seen that some pareto optimal solutions were better than the reference POP gene tree for both scores, suggesting that they could be of higher quality. As the true evolutionary histories of gene families are hardly known, relying on high-quality phylogenetic gene trees for biological analyses is preferable.

In summary, our results on the empirical dataset demonstrate how a GA framework can be used for the inference of gene trees with high accuracy.

## Conclusion

Algorithms for constructing gene trees from multiple sequence alignments are widely used. However when a reliable species tree is available, it is preferable to use species tree-aware methods which are often more accurate. In this work, we have presented a GA framework for the reconstruction of gene trees using both sequences and species tree information. From the comparison with existing methods, we have shown that this framework, implemented in a software called GATC, outputs more accurate gene trees.

As the true evolutionary history of a gene family does not always correspond to the most parsimonious one, GATC assumes instead that the true gene tree can most likely be found in the pareto optimal set of the search space. Therefore, given enough time, the algorithm will converge to a set of candidates containing that tree. Although this hypothesis was supported by our results on the empirical dataset, it does not necessary hold for all gene families. For example, since our reconciliation model does not consider Incomplete Lineage Sorting (ILS), the efficiency of GATC is expected to decrease in presence of ILS. Indeed, signals of deep coalescence leading to incongruence between species and gene tree would be explained by DTL events, possibly resulting in incorrect trees. Moreover, another problem still persists when there are several trees in the final pareto set, as alternative criteria for discriminating between these equivalent candidates are required. In its current implementation, GATC outputs solutions sorted by either the sequence likelihood or the reconciliation score.

Despite the good results we obtained by using GATC, one fundamental aspect that should be adressed in order to improve efficiency is the required evolution time. Indeed, running time cannot be accurately estimated especially when the starting trees have poor quality. When ML or bayesian trees have been inferred beforehand, it may be appropriate to set the maximum evolution time to the time required to find the best ML tree. As the underlying idea behind GAs allows for easy parallelism, running time can be dramatically reduced. Balance between scalability to large datasets and search efficiency would likely be achieved by carefully selecting the different genetic operators and the stopping criteria. Finally, to avoid being trapped in local optima, multiple replicate searches, using different settings (such as DTL, crossover and mutations rates, population size and initialization) can be performed in parallel with exchange of information through a migration operator.

## Additional file

Additional file 1 Contains supplementary information on the effect of operator rates (**Figure S1**) and errors in the species tree (**Figure S2**) on reconstruction accuracy. It also contains the original reference tree of the Poyeye family (**Figure S3**) and the four alternative trees obtained by GATC (**Figure S4-S7**). (PDF 315 kb)

## Abbreviations

AU: Approximately unbiased test
DL: Duplication-loss
DTL: Duplication-Transfer-Loss
FN: False negative
FP: False positive
GA: Genetic algorithm
GATC: Genetic algorithm for gene tree construction
HGT: Horizontal gene transfer
ILS: Incomplete lineage sorting
JTT: Jones, Taylor and Thornton substitution matrix
LG: Le and Gascuel substitution matrix
ML: Maximum likelihood
MOOP: Multi-objective optimization problem
MPR: Most parsimonious reconciliation
NSGA: Non-dominated sorting genetic algorithm
SPR: Subtree pruning and regrafting
TP: True positive
