# Supplementary material for: GATC: a genetic algorithm for gene tree construction under the Duplication-Transfer-Loss model of evolution
Source: BMC Genomics. 2018 May 9;19(Suppl 2):102. doi: 10.1186/s12864-018-4455-x (PMC5954287; doi:10.1186/s12864-018-4455-x)
Supplement: Supplementary file 1 — Contains supplementary information on the effect of operator rates (Figure S1) and errors in the species tree (Figure S2) on reconstruction accuracy. It also contains the original reference tree of the Poyeye family (Figure S3) and the four alternative trees obtained by GATC (Figure S4-S7). (PDF 315 kb) [file 12864_2018_4455_MOESM1_ESM.pdf]

Supplementary Materials for "GATC: A Genetic Algorithm for gene Tree Construction under the Duplication-Transfer-Loss model of evolution"

Emmanuel Noutahi[1,*] and Nadia El-Mabrouk[1]

[1]Département d'Informatique et de Recherche Opérationnelle, Université de Montréal
[*]To whom correspondence should be addressed; E-mail: fmr.noutahi@umontreal.ca

1 Effect of crossover and mutation rates on reconstruction accuracy

The performance of a genetic algorithm is strongly affected by the selected crossover and mutation rates. Moreover, the optimal crossover and mutation rates usually vary with the problem of concern. In order to measure the effect of these rates on GATC's reconstruction accuracy, we compared GATC results on a subset of 100 gene families from the simulated Cyanobacteria dataset under different rates of crossover (P_{cross}) and mutation (P_{mut}). We fixed the population size to 20, with a maximum number of generation set to 100 and used default parameters (LG + Gamma, $\lambda = 2$, $\tau = 3$, $\delta = 1$). In total, 15 different rates of crossover and mutation were evaluated : $(P_{cross}, P_{mut}) \in \{0.2, 0.4, 0.6, 0.8, 1\} \times \{0, 0.5, 1\}$. Trees of the initial population were obtained using PolytomySolver. We measured accuracy according to the error:

$$\Delta_E = \frac{\sum_i^N nRF(G_i, G_{true})}{N}$$

which corresponds to the mean normalized Robinson-Foulds distance between the gene trees of the last generation of evolution and the true phylogenetic tree of each simulated gene family.

A general tendency observed from the results depicted in Figure S1 is the decrease in the mean error for increasing rate of crossover. Similarly, for the same crossover rate,

higher rates of mutation usually yield better trees. Given the elitist nature of GATC's algorithm, it is expected that high crossover rates would likely result in faster convergence. There is however a risk of being stuck in a local optimum due to loss of diversity in the latter generations. The use of high mutation rates can partly help solve this problem, but it can also prevent the solutions from converging. For the comparison between GATC an other methods we use $(P_{cross}, P_{mut}) = (0.8, 0.5)$, which while being conservative, gave good results.

2 Robustness to errors in the species tree topology

Since integrative methods for gene tree reconstruction relies on the accurate reconciliation between gene and species trees, they are sensitive to errors in the species tree [2, 1]. To measure the effect of errors in the species tree on reconstruction accuracy, we considered five alternative topologies $(S_1, S_2, S_3, S_4, S_5)$ with increasing RF distance towards the assumed true Cyanobacteria species tree (S_{true}). These alternative topologies were obtained by performing SPR moves on S_{true}. We run six instances of GATC using a different species tree for the reconciliation framework each time, on 100 gene famillies. Trees of the initial population were obtained from bootstrap replicates and the same parameters as above were used with $(P_{cross}, P_{mut}) = (0.8, 0.5)$. We measure performance according to the mean error Δ_E between the tree of the last generation and the true simulated gene tree. A comparison between GATC's results on each of the six instances and RAxML trees is shown on Figure S2. As expected, GATC's performance is influenced by the error rates in the species tree, with alternative species tree that are most similar to S_{true} giving better results. GATC's average solutions were even more accurate than RAxML trees for species tree with only a few topological errors. Although species tree-aware methods are dependent upon the quality of the species tree, they can generally robust against uncertainties on only a few branches of the species tree. One possible approach to reduce the effect of unreliable species trees on gene tree accuracy is to build consensus gene tree with bootstrap values from multiple runs with alternative species tree topologies.

3 Supplementary Figures

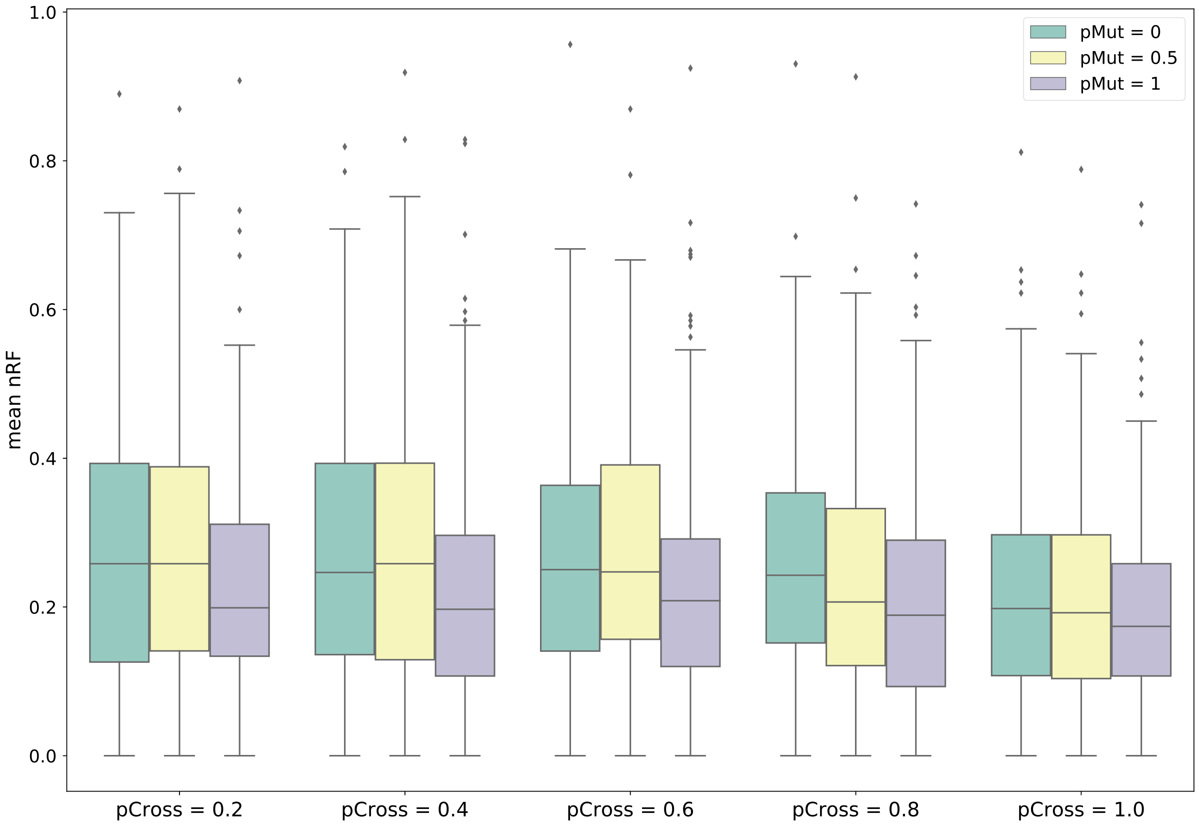

Figure S1: **Effect of various crossover and mutation rate on GATC's reconstruction accuracy**. A general tendency observed is the decrease in error rates for increasing rates of crossover and mutation.

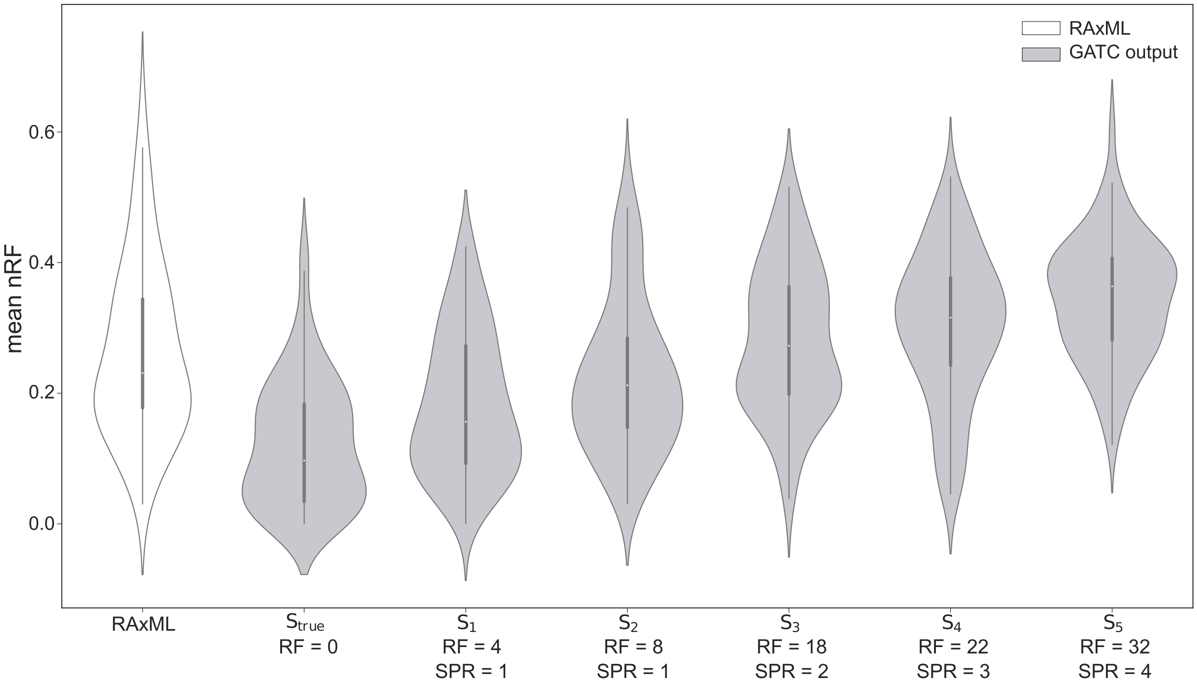

Figure S2: **Effect of using alternative species tree topologies on reconstruction accuracy.**. The accuracy of GATC's gene tree reconstruction decreases for increasing rate of errors in the species tree used. Nevertheless, the algorithm is robust to some extent against errors in the species tree topologies.

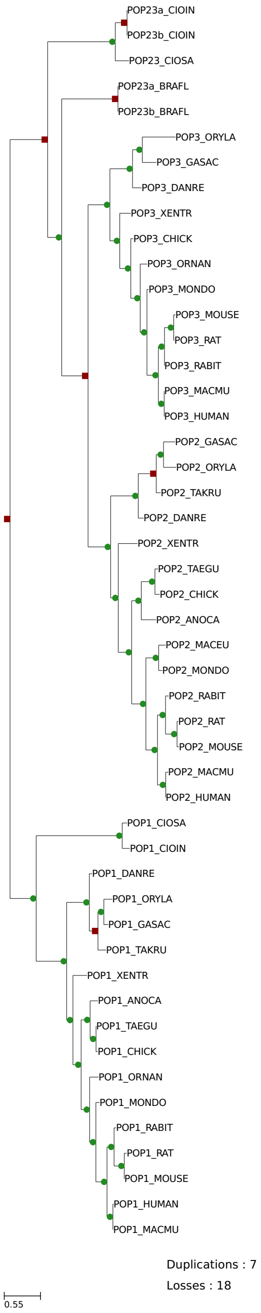

Figure S3: **Reference tree for the Popeye family.** Branch lengths are not shown. The gene tree was reconcilied with the species tree. Duplication nodes (leading to paralogs) are indicated by a red square, while speciation nodes(leading to orthologs) are indicated by a green circle. The total number of duplications and losses are shown at the bottom. Lost branches were not shown for clarity.

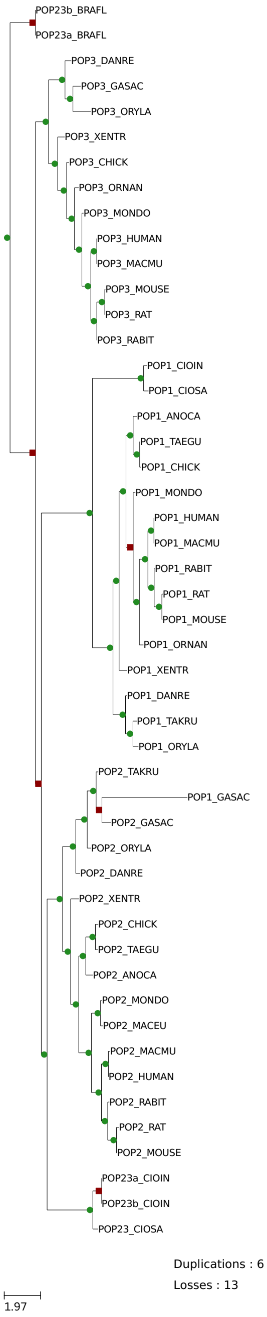

Figure S4: **Tree 1 return by GATC.** Branch lengths are not shown. The gene tree was reconcilied with the species tree. Duplication nodes (leading to paralogs) are indicated by a red square, while speciation nodes(leading to orthologs) are indicated by a green circle. The total number of duplications and losses are shown at the bottom. Lost branches were not shown for clarity.

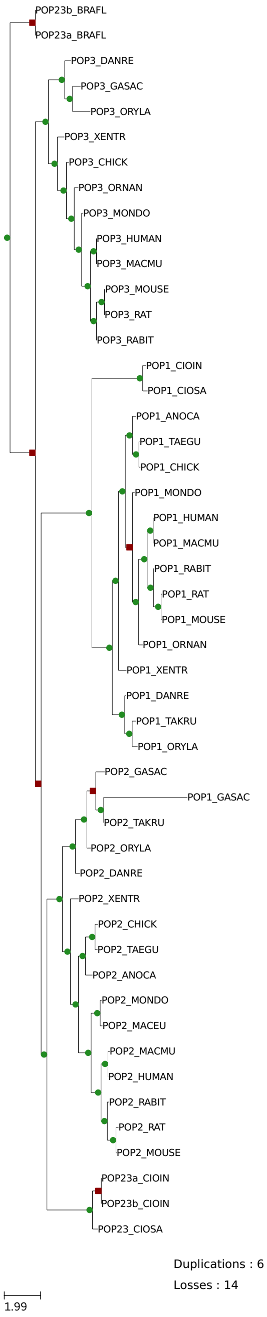

Figure S5: **Tree 2 return by GATC.** Branch lengths are not shown. The gene tree was reconcilied with the species tree. Duplication nodes (leading to paralogs) are indicated by a red square, while speciation nodes(leading to orthologs) are indicated by a green circle. The total number of duplications and losses are shown at the bottom. Lost branches were not shown for clarity.

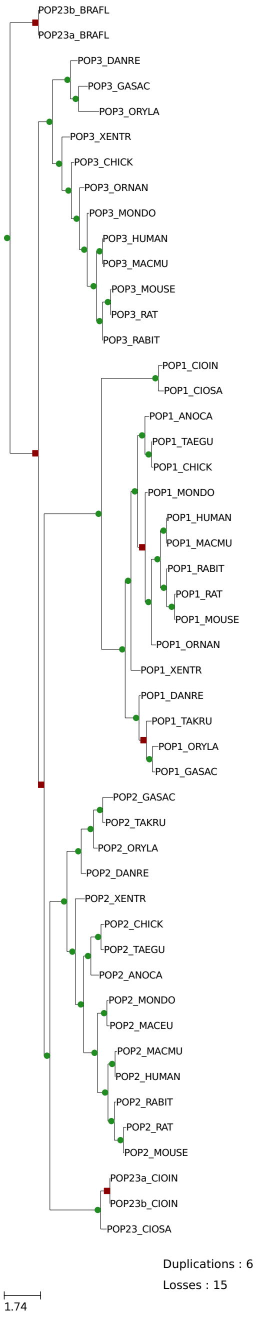

Figure S6: **Tree 3 return by GATC.** Branch lengths are not shown. The gene tree was reconcilied with the species tree. Duplication nodes (leading to paralogs) are indicated by a red square, while speciation nodes(leading to orthologs) are indicated by a green circle. The total number of duplications and losses are shown at the bottom. Lost branches were not shown for clarity.

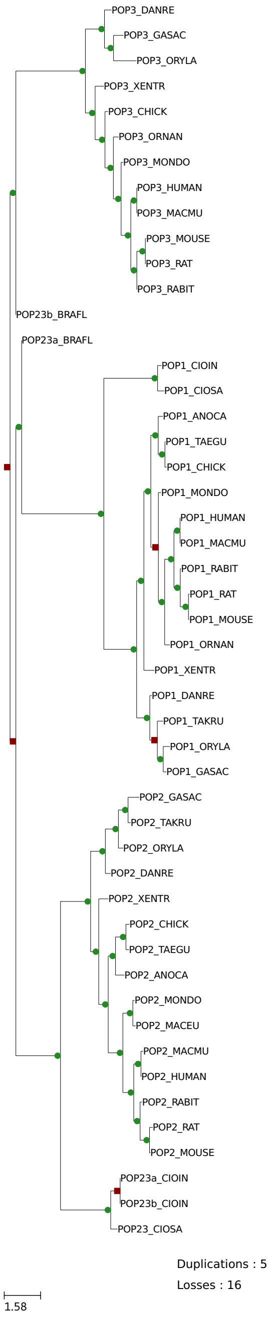

Figure S7: **Tree 4 return by GATC.** Branch lengths are not shown. The gene tree was reconcilied with the species tree. Duplication nodes (leading to paralogs) are indicated by a red square, while speciation nodes(leading to orthologs) are indicated by a green circle. The total number of duplications and losses are shown at the bottom. Lost branches were not shown for clarity.

References

[1] E. Noutahi, M. Semeria, M. Lafond, J. Seguin, L. Gueguen, N. El-Mabrouk, and E. Tannier. Efficient gene tree correction guided by genome evolution. *Plos.One*, 11(8), 2016.

[2] Y.C Wu, M.D. Rasmussen, M.S. Bansal, and M. Kellis. TreeFix: Statistically informed gene tree error correction using species trees. *Systematic Biology*, 62(1):110- 120, 2013.
